# Sexual Health Dysfunction After Radiotherapy for Gynecological Cancer: Role of Physical Rehabilitation Including Pelvic Floor Muscle Training

**DOI:** 10.3389/fmed.2021.813352

**Published:** 2022-02-03

**Authors:** Amelia Barcellini, Mattia Dominoni, Francesca Dal Mas, Helena Biancuzzi, Sara Carla Venturini, Barbara Gardella, Ester Orlandi, Kari Bø

**Affiliations:** ^1^Radiation Oncology Unit, Clinical Department, National Center for Oncological Hadrontherapy (CNAO), Pavia, Italy; ^2^Department of Clinical, Surgical, Diagnostic and Paediatric Sciences, University of Pavia, Pavia, Italy; ^3^Department of Obstetrics and Gynecology, Fondazione IRCCS Policlinico San Matteo, Pavia, Italy; ^4^Lincoln International Business School, University of Lincoln, Lincoln, United Kingdom; ^5^Center of Organization and Governance of the Public Administration, University of Pavia, Pavia, Italy; ^6^Ipazia, International Observatory on Gender Research, Rome, Italy; ^7^Department of Sports Medicine, Norwegian School of Sports Sciences, Oslo, Norway; ^8^Department of Obstetrics and Gynecology, Akershus University Hospital, Lorenskog, Norway

**Keywords:** gynecological cancers, pelvic floor muscle training, radiotherapy, rehabilitation, sexual health, vaginal toxicity

## Abstract

**Introduction:**

The present study aims to describe: 1. How the side effects of radiotherapy (RT) could impact sexual health in women; 2. The effectiveness of physical rehabilitation including pelvic floor muscle training (PFMT) in the management of sexual dysfunction after RT.

**Materials and Methods:**

Search keys on PubMed, Web of Science, Scopus, PEDro, and Cochrane were used to identify studies on women treated with radical or adjuvant RT and/or brachytherapy for gynecological cancers with an emphasis on vulvo-vaginal toxicities and PFMT studies on sexual dysfunction for this group of women.

**Results:**

Regarding the first key question, we analyzed 19 studies including a total of 2,739 women who reported vaginal dryness, stenosis, and pain as the most common side effects. Reports of dosimetric risk factors and dose-effect data for vaginal and vulvar post-RT toxicities are scant. Only five studies, including three randomized controlled trials (RCTs), were found to report the effect of PFMT alone or in combination with other treatments. The results showed some evidence for the effect of training modalities including PFMT, but to date, there is insufficient evidence from high-quality studies to draw any conclusion of a possible effect.

**Conclusions:**

Gynecological toxicities after RT are common, and their management is challenging. The few data available for a rehabilitative approach on post-actinic vulvo-vaginal side effects are encouraging. Large and well-designed RCTs with the long-term follow-up that investigate the effect of PFMT on vulvo-vaginal tissues and pelvic floor muscle function are needed to provide further guidance for clinical management.

## Introduction

During the past two decades, advances in oncology, radiotherapy (RT), and surgery have significantly improved the survival rate of women affected by gynecological cancers (GyC) (1–6). In recent years, there has been an emerging interest in late treatment sequelae. Vulvodynia (7), dyspareunia (8, 9), fecal and urinary incontinence (10), lower limb lymphedema (11), hot flashes (12), fatigue (13), insomnia (12), and emotional distress (14) are frequently reported side effects that could considerably affect the quality of life (QoL) of GyC survivors (4, 15). Moreover, pelvic RT can lead to premature menopause in young women or to a worsening of menopausal syndrome with increased metabolic, cardiovascular, and osteoporotic risks (16). Not only women who underwent RT but also patients with ovarian cancers (17) experienced a reduction of sexual function after diagnosis and oncological treatment. However, several of these side effects appear as underreported and undertreated (18, 19) even though more than 40% of cancer survivors expressed interest in receiving sexual healthcare (20). Understanding and treating physical symptoms and the consequent psychological issues stand as primary challenges for the healthcare staff dealing with GyC survivors. RT studies usually describe radiation-induced organ-related morbidity (particularly for bladder, rectum, and bowel toxicities) with the aim to improve the dose optimization approach. Despite the impact on QoL of long-survivors and sexually active patients, the vagina has so far only slightly been included in the panel of organs at risk (OARs) in the RT planning treatment for GyC. To date, there is no consensus about vaginal dose constraints (19). The psychological stress of sexually related toxicities negatively impacts the QoL of GyC survivors who often report feelings of shame, inadequacy, emotional distancing from the partner, and alteration in body image (21–23).

Several studies have reported that external beam RT (EBRT) and brachytherapy (BT) could significantly affect the pelvic floor muscles due to the development of fibrosis in the smooth and the striated layer of the muscle tissue (24, 25) and, thus, lead to urinary incontinence, anal incontinence, and sexual distress. The long-term side effects of RT cause an alteration of vaginal structure, such as vaginal stenosis, conglutination, dryness, and dyspareunia. The pathological process of vaginal tissue damage appears as a decrease in vaginal length, in the elasticity of the muscles layer, and vaginal lubrication (26, 27). Meta-analysis, systematic reviews, and clinical trials have found that physical activities are effective in decreasing the state of systemic inflammation, reducing cancer-related-fatigue, and improving current QoL (28–36), but there are still few reports about rehabilitation and pelvic floor muscle training (PFMT) to alleviate vaginal and vulvar symptoms due to RT toxicities (37–39). This systematic review aims to evaluate:

- How the side effects of RT could impact the sexual health in women.
- The effectiveness of physical rehabilitation, including PFMT in the management of sexual dysfunction after RT.

## Materials and Methods

### Data Sources and Searches

A comprehensive literature search on the research questions was conducted during April 2021. PubMed, Web of Science, PEDro, and Scopus (40) were searched for published studies.

For studies on sexual health morbidity after RT in GyC survivors, the database search was done with a combination of the following keywords: “pelvic radiotherapy,” “toxicity,” “vaginal toxicity,” “vaginal brachytherapy,” “hadrontherapy,” “radiotherapy,” “rehabilitation,” “gynecological cancer,” “sexual health,” “quality of life”, “sexual dysfunction,” “pelvic floor,” including pluralization and US English/UK English spelling variations and suffixes/prefixes.

For studies on physical therapy, the search was performed with a combination of the following words: “pelvic floor,” “pelvic floor muscle,” “physiotherapy,” “training,” “exercise,” “education,” “dilator,” “physical therapy,” “pelvic radiotherapy,” “gynecological cancer,” “brachytherapy,” “toxicity,” “cancer,” “tumor,” including pluralization and US English/UK English spelling variations and suffixes/prefixes.

### Study Selection and Data Extraction

We defined inclusion criteria for the literature search using the Population, Intervention, Control, Outcome, and Study (PICOS) design approach (41).

#### Patient Populations of Interest

We included studies of women with GyC treated with RT and/or BT, with or without concomitant chemotherapy in adjuvant and radical settings. We omitted studies considering re-irradiation or palliative RT.

#### Intervention and Control

The intervention for the first question was BT or/and RT as definitive or adjuvant therapy. We organized studies for our analysis considering the RT delivered (BT, EBRT, carbon-ion RT -CIRT-). We considered BT both employed as monotherapy or in combination with EBRT. The intervention for the second question was PFMT and physical activities aimed at reducing gynecological RT toxicities in women who had undergone RT for GyC (e.g., PFMT, vaginal massage, or dilator training). For PFMT, we considered a program of “repeated voluntary PFM contractions taught and supervised by a healthcare professional” (for example, PFMT for strengthening or relaxation, for urge suppression, single contractions to instantly control/prevent leakage) (42, 43). Individual or group PFMT, or relaxation training, with or without biofeedback were included.

#### Outcomes of Interest

The primary outcome measure for the first research question was that vaginal and vulvar post-RT toxicities were scored according to the Common Terminology Criteria for Adverse Events (CTCAE) scale (44), Radiation Therapy Oncology Group (RTOG) (45), and Dische score (46). Vulvodynia, when available, was assessed according to the ISSVD (International Society for the Study of Vulvovaginal Disease), ISSWSH (Boards of Directors of the International Society for the Study of Women's Sexual Health), and IPPS (International Pelvic Pain Society) Consensus Conference classification (47). Table 1 summarizes the vulvo-vaginal toxicities according to the CTCAE scoring system (44). For the second research question, PFM function, pain, and QoL were reported.

**Table 1:** Vulvo-vaginal morbidity after radiotherapy: definition and score according to Common Terminology Criteria for Adverse Events (CTCAE) v 5.0.

**Endpoint**	**Definition**	**Grade 1**	**Grade 2**	**Grade 3**
Vaginal Pain^*^	Mild pain	Moderate pain; limiting instrumental ADL	Severe pain; limiting self-care ADL	
Dyspareunia	A disorder characterized by painful or difficult coitus.	Mild discomfort or pain associated with vaginal penetration; discomfort relieved with use of vaginal lubricants or estrogen	Moderate discomfort or pain associated with vaginal penetration; discomfort or pain partially relieved with use of vaginal lubricants or estrogen	Severe discomfort or pain associated with vaginal penetration; discomfort or pain unrelieved by vaginal lubricants or estrogen
Vaginal dryness	A disorder characterized by an uncomfortable feeling of itching and burning in the vagina.	Mild vaginal dryness not interfering with sexual function	Moderate vaginal dryness interfering with sexual function or causing frequent discomfort	Severe vaginal dryness resulting in dyspareunia or severe discomfort
Vaginal Stenosis	A disorder characterized by a narrowing of the vaginal canal.	Asymptomatic; mild vaginal shortening or narrowing	Vaginal narrowing and/or shortening not interfering with physical examination	Vaginal narrowing and/or shortening interfering with the use of tampons, sexual activity or physical examination

*ADL, Activities of Daily Living*.

* *CTCAE v 5.0 doesn't report vulvar pain/vulvodynia*.

Study designs for the first research question were prospective cohort studies, cross-sectional studies, case-control, retrospective studies, and case series. Single case reports were excluded. For the second research question, both randomized controlled trials (RCTs) and uncontrolled trials were included. Case studies were excluded. In the case of duplicated datasets (e.g., multiple articles from the same study group or institution, related to the same treatment on the same cohort of the patient), only the manuscript with the most extended follow-up and the largest cohort was included.

### Data Extraction and Quality Assessment

We screened the data, which included author names, publication year, study design characteristics, number of patients, age, histology, radiation technique and dose (total and for fraction), reported vaginal and vulvar toxicity, toxicity scale used, and follow-up time. For the second research question, we also extracted the intervention program type, duration, frequency, intensity, supervision, adherence, dropout, and outcomes.

### Data Synthesis and Analysis

**Figure 1:**
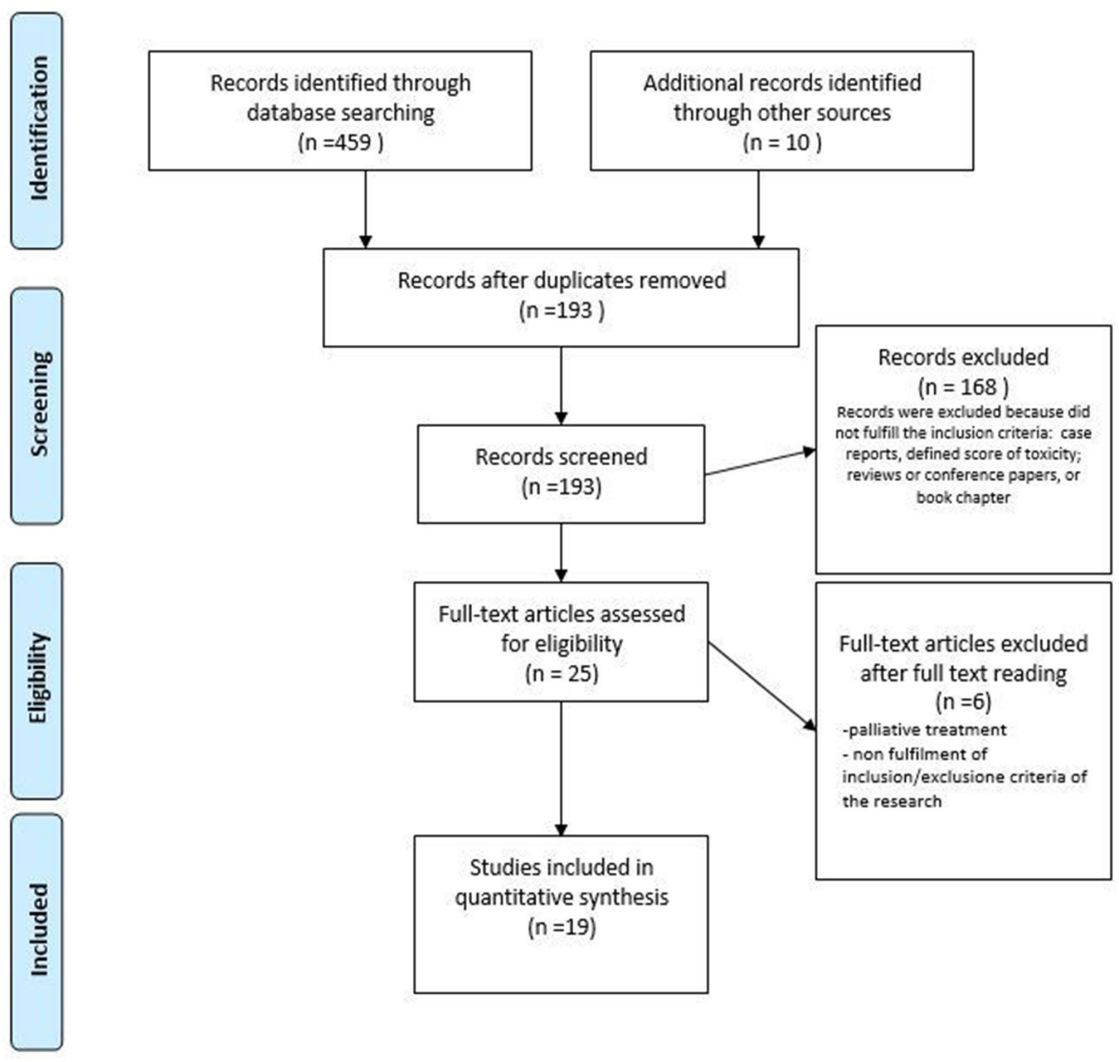
PRISMA flow diagram of the study selection process for vulvar and vaginal radiotherapy toxicity. Adapted from (48).

**Figure 2:**
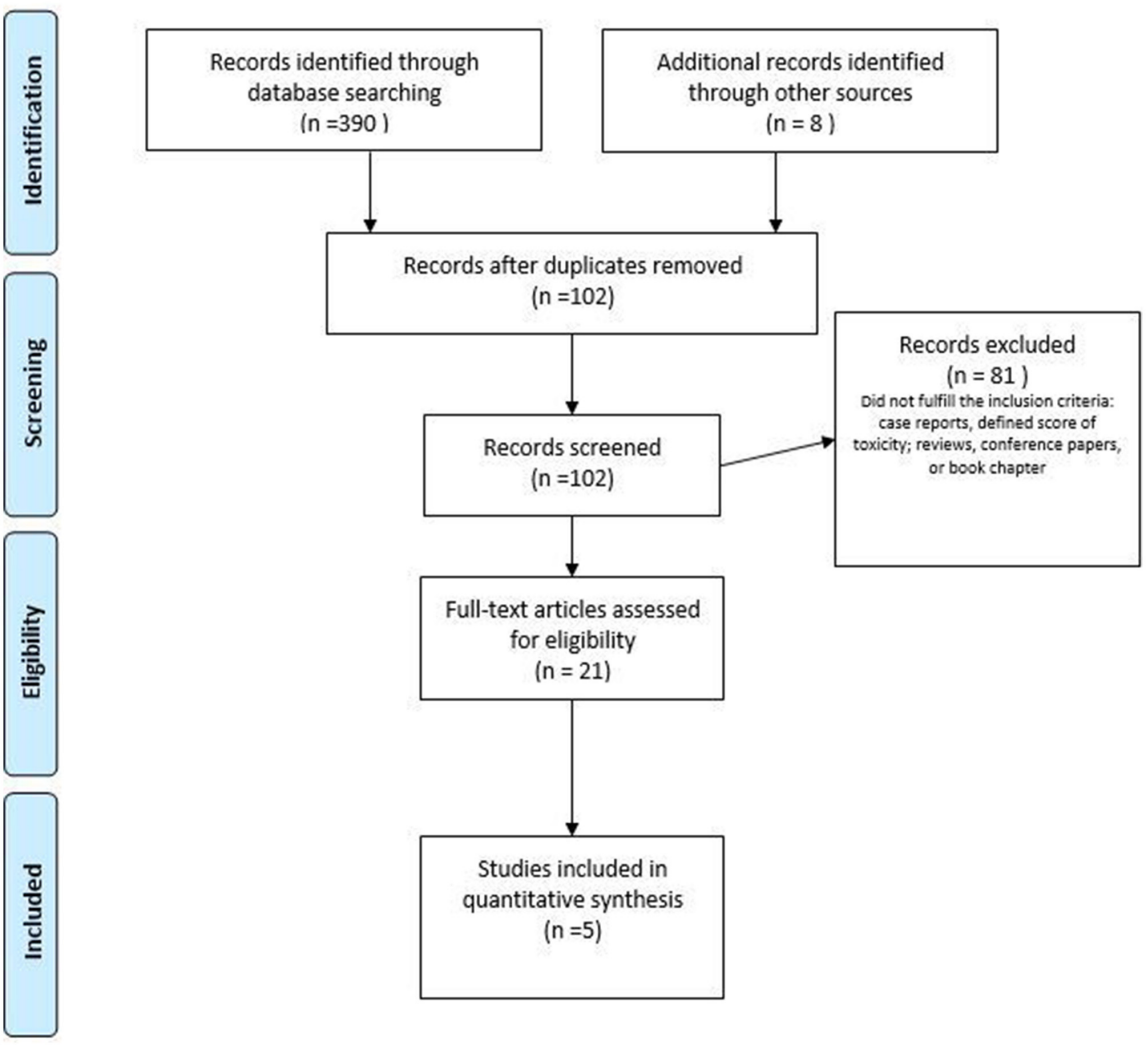
PRISMA flow diagram of the study selection process for pelvic floor muscle training **(PFMT)** as conservative management of sexual dysfunction after radiotherapy **(RT)**. Adapted from (48).

The flowcharts of the two literature analyses are displayed in Figures 1, 2. Studies are organized into two tables (Tables 2, 3). The RT toxicities analysis follows a descriptive analysis. We reported a descriptive analysis for the rehabilitation approach, and for RCT, the methodological quality of the studies was evaluated using the PEDro scale (Table 3). The PEDro method is a checklist of 10 items assessing the internal validity of clinical trials and 1 item assessing the external validity. The maximum possible score is 10/10 (excluding external validity item), with scores of ≥7 indicating high-quality study designs, while scores of 5–6 indicating moderate-quality study designs, and scores of <5 indicating low-quality study designs (70).

**Table 2:** Radiotherapy toxicities in the analyzed studies.

**References**	**No of pts**	**Tumor localization**	**Age (mean ad range)**	**RT technique**	**RT aim**	RT Doses/fraction (*)	**Follow-up (median)**	**Late vaginal toxicity**	**Type of toxicity**	**Score used**
MacLeod et al. (49)	143	Endometrial cancer	62 (38–90)	BT	Adjuvant	34 Gy/4	29 months	15.4% G1–2	Discharge	RTOG/EORTC
Onsrud et al. (50)	217	Endometrial cancer	61 (32–85)	BT	Adjuvant	22 Gy/4	84 months	24.4% G1–2	Stenosis	Chassagne
Greven et al. (51)	46	Endometrial cancer	NR	BT	Adjuvant	18Gy/3	29 months	26.1% G1–2	NR	RTOG/EORTC
Sorbe et al. (52)	290	Endometrial cancer	64.5 (40–89)	BT	Adjuvant	15–30 Gy/ 6	60 months	24.6% G1–2	Discharge, Dryness, Bleeding, Itching	NR
Chong et al. (53)	173	Endometrial cancer	64 (36–91)	BT	Adjuvant	22 Gy / 4	38 months	12.7% G1–2	Stenosis, Bleeding	CTCAE 3.0
Sorbe et al. (54)	319	Endometrial cancer	68 (41–88)	BT	Adjuvant	18−24 Gy /3–6	60 months	7.5% G1–2, <1% G3–4	Stenosis, Fibrosis, Athrophy, Bleeding	NR
Rovirosa et al. (55)	112	Endometrial cancer	66 (39–90)	BT	Adjuvant	10–30 Gy/2–4	30 months	24.9% G1–2, <1% G3–4	Stenosis	RTOG/EORTC
Landrum et al. (56)	23	Endometrial cancer	69 (46–81)	BT	Adjuvant	21 Gy/3	36 months	13.1% G1–2	Dyspareunia, Stenosis, Dryness	CTCAE 4.0
Laliscia et al. (57)	126	Endometrial cancer	67 (27–90)	BT	Adjuvant	21 Gy/3	29 months	23% G1–2	Fibrosis, Stenosis, Dryness	CTCAE 4.2
De Sanctis et al. (58)	108	Endometrial cancer	65 (35–86)	BT	Adjuvant	21 Gy/3	44 months	3% G1–2	NR	RTOG-LENTSOMA
Qian et al. (59)	304	Endometrial cancer	65 (43–94)	BT	Adjuvant	14–21 Gy/2–3	18 months	16.7% G≥1	Stenosis	CTCAE 4.0
Barcellini et al. (8)	14	Vaginal intraepithelial neoplasia grade 3 (VAIN3)	60.5 (43–77)	BT	Radical	24–42 Gy/4–7	32.7 months	35.7% G2; 21.4% G3	Stenosis	CTCAE 4.0
Blanchard et al. (60)	28	Vaginal intraepithelial neoplasia grade 3 (VAIN3)	50 (29–78)	BT	Radical	60 Gy/ 0.4–0.6 Gy/hour	41 months	25% G1	Dyspareunia, Teleangectasia, Shortening	CTCAE 4.0
Graham et al. (61)	22	Vaginal intraepithelial neoplasia grade 3 (VAIN3)	56 (37–71)	BT	Radical	22–26 Gy/145–205 cGy/h	77 months	100% G1; 18.18% G3, 5.5 G4	G1 dryness, teleangectasias; G3 stenosis, G4 vaginal ulcera	RTOG/EORTC
Song et al. (62)	34	Vaginal intraepithelial neoplasia grade 3 (VAIN3)	53 (33–71)	BT	Radical	40 Gy/8	48 months	29.41% G1–2; 5.88% G3	G1–2 infammation, stenosis; G3 stenosis, dyspareunia	CTCAE 4.0
Laliscia et al. (1)	56	Vulvar cancer	72 (37–91)	EBRT ± BT	Salvage radiotherapy for recurrence	EBRT group: 45–70 Gy/28–35; Interstitial BT: 31.5–51 Gy BID; Intracavitary BT: 21 Gy/3 fx	35 months	EBRT group: 7.0% G2; 9.3% G1–2 BT group 7.7% G3	EBRT group: G2 vaginal fibrosis G1–2 vaginal stenosis; BT group G3 stenosis	CTCAE 4.0
Kirchheiner et al. (63)	630	Cervical cancer	49 (22–89)	EBRT+BT	Radical	45–46 Gy EBRT + HDR/PDR BT	24 months	41% G1; 17%G2, 1% G3	Stenosis	CTCAE 3.0
Yoshida et al. (46)	57	Cervical cancer	59 (30–88)	EBRT+BT	Radical	50.4 Gy + 16.5–47 Gy interstitial BT or 30–36 endocavitary BT	36 months	53% G ≥2 stenosis rates at 3 years (69% at 5 years), 100% G ≥2 stenosis rates at 3 years with G≥2 pallor reactions at 6 months; patients with grade ≥2 pallor reactions G ≥2 stenosis rate (100% at 3 years or later)	Stenosis, Pallor reactions	Dische score, LENTA SOMA
Murata et al. (64)	37	Malignant gynecological melanoma	71 (51–88)	CIRT	Radical	57.6–68.8 GyE/16	23 months	24.32% G1	Dermatitis/Mucositis	CTCAE4.0

**Table 3:** Pelvic floor muscle training (PFMT) alone or in combination with other treatments for sexual dysfunction in gynecological cancer (GyC) patients.

**References**	**Study design**	**Tumors**	***N***.	**Treatment for GyC**	**Start of intervention**	**PFM approach**	**Outcomes**	**Bias**	**Pedro score**
Robinson et al. (65)	Randomized controlled trial	Endometrial cancer Cervival cancer	32 1.PFMT group: (*n* = 18) 2. no-PFMT group (control): *n*: 14)	PFMT group vs. control RT alone 6+3 RT+ CT: 2+6 surgery+ RT: 7+5 surgery + RT and CT: 3+0	After the beginning of RT and in the follow-up setting	Psychoeducational program: Information about sexuality, instruction for the use of lubricants and dilatation Motivation: promote sexuality despite treatment Behavioral skills: teaching to use dilatators and lubricants and perform PFMT (Kegel exercises)	Evaluation of Sexual History Form score: no difference between baseline scores and after intervention Evaluation of sexual knowledge questionnaire: effect of the intervention depended on the age of the subject -Evaluation of fears: intervention reduce fears, and fear scores were 0.333 points lower in the intervention group (95% CI [0.051-0.615]) -Evaluation dilation compliance: effect of the intervention depended on the age	Small sample size Short follow-up No details about PFMT (Kegel exercises)	4
Yang et al. (66)	Randomized controlled trial	Endometrial cancer Cervical cancer	28 1.PFMT group: (*n* = 14) 2.no-PFMT group (control): (*n* = 14)	PFMT group vs. control Surgery only 1 + 1 Surgery+ RT 4+4 Surgery+CT 6+ 5 Surgery +RT +CT 3+ 4	Follow-up Setting (after oncological treatment)	PFMT group: 45-min exercise session and 30-min counseling for each session per week for 4 weeks 20 min-Biofeedback: lithotomy position *via* a vaginal silicon pressure device. 20-min- Transverse abdominal training associated with Diaphragmatic breathing techniques and stretching exercises of the hip muscles: Home exercises: 10 maximum voluntary PFM contractions held for up to 10 s, with a 4-s rest between contractions, and followed by 1-min rest and 10 or more fast contractions for 20–30 s.	Significant improvement in PFM strength (*P* = 0.036) and sexual functioning (*P* = 0.048) for the PFM training group Mean difference (MD) between groups (intervention vs. control) improvements in PFM strength (MD = 14.22; *P* = 0.036). - lower excitability threshold to cortical stimulation (%), (MD= −20.29; *P* = 0.014)	Small sample size Short-term pilot study Short duration and low intensity of the PFM training.	5
Bakker et al. (67)	Pre-post test intervention	Endometrial cancer Cervical cancer Vaginal cancer	20	EBRT/BT	Follow up setting (after oncological treatment)	Education: Information by nurses about vaginal dilators, lubricants, information booklet about sexual rehabilitation (no details of the content reported)	Improvement in sexual functioning measured by FSFI (*p* < 0.001) and frequency of vaginal dilation and intercourse.	Small sample size -Not randomized control group -Dropout rate of 40% [-] PFMT not specified	N/A
Lubotzky et al. (68)	Randomized controlled trial	Endometrial cancer Cervical cancer Vulvar cancer Vaginal cancer Anorectal cancer	82 1. PFMT group (*n* = 44): received a PFMT booklet 2. Control (*n* = 38): received standard information material	RT (46) BT (38) CT (39) Surgery (55)	Follow-up Setting (after oncological treatment)	PFMT received a booklet about the use of vaginal dilatator+ PFM training+ Lubricant/ moisturizer no further details of the PFMT protocol are available	Improvement in dilator adherence (*p* < 0.01) but not in PFMT and the use of lubricants/moisturizers About PFMT the point estimates and variation not reported No report of sexual dysfunction or PFM function	No details about PFMT Selection bias Short follow-up.	5
Cyr et al. (69)	Pre-post test multicenter intervention	Endometrial cancer Cervical cancer	31	Surgery 24 BT 19 RT 15 CT 16 Current use of menopausal hormone therapy 4	Follow-up setting (after oncological treatment)	12 weekly individual 60 min sessions: Education (about pathophysiology and management of dyspareunia, use of vaginal lubricant and moisturizer) 20–25 min of manual therapy (stretching, myofascial release, pressure and massage) applied externally and intravaginally to the PFM by physical therapists. From the 7th to the 12th session: vestibule massage and desensitization 20-min PFMT with biofeedback (using a small intravaginal probe to foster PFM relaxation, coordination, strength and endurance). 5 times weekly Home exercises with biofeedback (PFM for relaxation, coordination, strength and endurance)+ deep breathing+ contraction exercises) -Vaginal dilatator exercises (introduction, relaxation, clock stretching, oscillation)	Significant improvement in all outcomes (*p* ≤ 0.0044), variation express in mean ±SD -Reduction in pain intensity (NRS −5.6 ± 2.24) -Reduction in pain quality (MPQ −12.9 ± 14.7) -Improvement in sexual functioning (FSFI 6.9 ± 6.4) Improvement of sexual activities with vaginal penetration 1.6 ± 1.9 per month)	Not randomized control group Sample size	N/A

## Results

### Key Question 1: How Could the Side Effects of RT Impact Sexual Health in Women?

While the initial results led to 193 publications, after the screening process, the final sample included 19 studies. One prospective and 18 retrospective cohort studies were included, and the study characteristics are shown in Table 2. A total of 2,739 women were included in the studies, with 630 in the prospective cohort studies. The age of the patients across all studies ranged between 22 and 94 years. Endometrial and cervical cancers were the most frequently represented tumors. The majority of patients underwent BT alone (1,959 patients, 71.5%), followed by EBRT with or without a BT boost (743 patients, 27.13%) and a CIRT approach (37 women, 1.30%). The most common toxicity scale used was CTCAE (71), followed by RTOG/European Organization for Research and Treatment of Cancer (EORTC) (49, 51, 55, 61), RTOG/ Late Effects Normal Tissues (LENT)-Subjective, Objective, Management, Analytic (SOMA) (46, 58) (Table 2). In two studies, toxicities were scored by Dische (46) and Chassagne scores (72). The most common vaginal toxicities reported after pelvic RT (for each RT technique) were stenosis, dryness, and dyspareunia. In the articles analyzed, vulvodynia was not scored by the above-reported classifications.

### Key Question 2: The Effectiveness of Physical Rehabilitation Including PFMT in the Management of Sexual Dysfunction After RT

With regards to GyC, five clinical trials (65–69) involving the role of PFMT were found, of which three were RCTs (Table 3). The number of participants ranged between 20 and 82. The duration of the intervention period ranged between 4 and 12 weeks, respectively, in the studies that reported the duration, frequency, and intensity of the training program. The interventions differed among studies and combined a plethora of modalities. The interventions ranged from supervised PFM strength and relaxation training combined with manual techniques (e.g., massage) to handling out a booklet with information or psychoeducational training. All the studies included vaginal device training. The one RCT reporting point estimates and variation between groups found that a multimodal PFMT intervention was statistically significantly superior to an untreated control group concerning PFM strength and sexual function (Table 3). The effectiveness of a psychoeducational approach seemed to depend on the age of participants as well as the dilation compliance (65). The PEDro score for the RCTs ranged between 4 and 5, for the analyzed studies (73) (Table 3).

## Discussion

The present systematic review found that post-actinic vulvo-vaginal toxicity in long-term survivors from GyC is mostly represented by dryness, stenosis, and dyspareunia. In the analyzed data, the authors rarely suggested the management of this specific chronic toxicity, and it is also interesting to highlight that sexual health is poorly reported in these RT studies.

Regarding dosimetric studies, data related to vaginal and vulvar toxicities are scant (8, 63). Vulvar and vaginal tissues, currently vaguely included in the panel of OARs in RT planning, must be regarded due to the consequent morbidity (19).

Modern radiation techniques such as hadrontherapy are promising (5, 6, 74–76), but our review shows that data is scant both about dosimetric strategies to reduce vaginal toxicities and the radiation effect of PFM structures. These limitations highlight the need for further high-quality research.

The lack of data on RT and dosimetric studies is interesting considering that, in the PEDro database, with a combination of the following keywords “rehabilitation,” “pelvic floor muscle,” and “cancer,” we have found 24 records about rehabilitation in prostatic cancer, including one practice guidelines, seven systematic reviews, and 16 clinical trials. This is coherent with the results of the “igls-Vienna-sexmed-survey” (77) in which radiation oncologists showed higher awareness regarding male compared to female sexual functioning. Most radiation oncologists are not experts in treating sexual dysfunction (77), and more specific training seems of utmost importance to improve the attitudes and behavior toward sexual issues of GyC patients (78).

Usually, the main recommendations to women at the end of RT delivered to the pelvis with or without vaginal and/or endouterine BT are to resume sexual activity or to avoid the collapse of the vaginal walls with the use of vaginal dilators (79, 80). Unfortunately, this approach is often poorly tolerated by women with low adherence and compliance, often depending on the age of patients (65). Although in most studies the use of vaginal dilator was encouraged to reduce vaginal toxicity, Brennen et al. (81) reported a “very low” level of evidence of this approach, according to the Grading of Recommendations Assessment, Development, and Evaluation (GRADE) analysis (82). Studies show that specific educational training also through a tailored-booklet proved to be effective in guaranteeing greater adherence and improving sexual health, especially in younger patients (65, 67, 68).

Data derived from trials based on men treated for prostate cancer in acute and long**-**term conditions revealed that there was no difference between pre- and post-radiation therapy in maximal thickness of external and internal anal sphincter (83–85). For men, RT toxicities after prostatic cancer are also reported to change the levator ani muscles and urogenital diaphragm (85) as well as reduce the ureteral length, and modifying periureteral muscles and the periprostatic portion of levator ani muscles due to post-RT muscle fibrosis of muscles (83).

In their systematic review and meta-analysis, Brennen et al. (78) reported that a combined approach, including PFMT, counseling, and physical exercises (core training and yoga), significantly improved the sexual health outcomes of GyC survivors. PFM functions, especially muscle strength, play a fundamental role in sexual function. Women with high levels of pelvic floor muscle contractions on physical evaluations achieved higher scores on Female Sexual Function Index (FSFI) (86), and the improvement of PFM control is related to a reduction of dyspareunia (18). However, the above-reported analysis included several pelvic health outcomes (i.e., bladder and rectal functioning) including women who did not undergo RT.

From a physical point of view, RT decreases the force of pelvic floor muscles with a reduction of recruitment of motor units. The modification in muscle tissue histology influences the competence to create strength and force rapidly, which may be important in counteracting the increased intra-abdominal pressure (87–90). The post-actinic muscle fiber damages could also lead to a reduction of spontaneous muscle activity and contractile response to stimulation (91) with a decrease in the ability to perform maximal strength rapidly (muscle power) as well as to maintain the same force in a series of repeated contractions (90, 92).

The rehabilitation programs aim to overcome the adverse effects of RT on pelvic floor muscles and to restore functionality in order to mitigate sexual distress, and bladder or bowel symptoms. The rehabilitation of women previously treated for pelvic cancers may decrease urinary incontinence and urgency, sexual dysfunction and discomfort, and improve the quality of life due to effective restoration of strength and an increase of blood flow in the pelvic floor tissue (66, 90, 93). However, our literature review found only 3 RCTs, and they had huge heterogeneity of populations, interventions, and use of outcome measures. The RCTs scored low to moderate on the PEDro score, with lack of blinding, loss to follow-up, and intention to treat analysis compromising the internal validity of the results. As the studies also included many different approaches to rehabilitation, it is not possible to conclude whether PFMT alone has a role in the rehabilitation of sexual function in GyC survivors.

In their RCT, Yang et al. (66) found that PFMT combined with core training improved pelvic strength and perceived sexual functioning leading to a significant increase in the proportion of sexually active women. However, whether core training has a role in the rehabilitation of PFMT has been debated, so far there is no evidence for this intervention alone or in combination with PFMT for urinary incontinence (94, 95) or sexual dysfunction (96).

The potential preventive role of routine use of vaginal dilators and the level of evidence about this practice after RT are still not clear (79, 81). A Cochrane review (79) reported no dependable evidence to consider that routine and constant use of vaginal dilators during RT prevents vaginal stenosis, although this practice is associated with lower rates of self-reported stenosis. The GRADE analysis by Brennen et al. (81) showed a “very low” level of evidence for the decrease in vaginal complications with the high use of vaginal dilators.

A multimodal approach with PFMT and the use of vaginal moisturizers seem feasible for GyC survivors (69). Hyaluronic acid seems to be effective and safe in the treatment of vaginal acute and late RT toxicities (97). Despite the controversial carcinogenesis risk of hormone replacement therapy and the lack of high-level evidence (98) in their systematic review, Vargiu et al. reported the benefits of this approach for the management of early menopause in patients with cervical cancer (16).

## Conclusion

Due to few data, large heterogeneity, and the low methodological quality of the included studies in our review, results should be interpreted with caution; however, our findings indicate that the rehabilitation approach (including PFMT and vaginal dilator training) may be effective and feasible in improving sexual function and in GyC patients who have undergone RT. To improve our knowledge and evidence for clinical practice of GyC survivors, we suggest a multidisciplinary approach between oncologists (radiation oncologist, medical oncologist, gynecological oncologists) and experts in rehabilitation and physical therapy in addressing the following research questions:

- What is the effect of RT with or without BT on the female PFM?
- What is the effect of PFMT in women with post-RT vulvo-vaginal toxicity?
- Does the effect of PFMT on vulvo-vaginal symptoms differ according to the total RT dose, fractionation schedule, and type of RT?
- Does preventive PFMT, before and during RT, improve sexual functioning?
- Is PFMT cost-effective in GyC patients?

Large, well-designed RCTs with long-term follow-up, which explicitly measure adherence and investigate the effect on vaginal function are needed to answer these questions.

## Author Contributions

AB and KB contributed to the concept and research design. AB, MD, and SV contributed to data collection. AB and MD contributed to the writing. HB, FD, EO, and BG critically revised the manuscript. All the authors read and agreed to the published version of the manuscript.

## Conflict of Interest

The authors declare that the research was conducted in the absence of any commercial or financial relationships that could be construed as a potential conflict of interest.

## Publisher's Note

All claims expressed in this article are solely those of the authors and do not necessarily represent those of their affiliated organizations, or those of the publisher, the editors and the reviewers. Any product that may be evaluated in this article, or claim that may be made by its manufacturer, is not guaranteed or endorsed by the publisher.
